# Case report: Rare presentation of double primary malignancies of the lung and thyroid: a difficult diagnosis

**DOI:** 10.3389/fonc.2023.1251492

**Published:** 2024-01-08

**Authors:** Shun-Ping Chen, Peng Li, Yi-Fei Pan, Xin Jiang

**Affiliations:** ^1^Department of Ultrasonography, The First Affiliated Hospital of Wenzhou Medical University, Wenzhou, China; ^2^Department of Pathology, The First Affiliated Hospital of Wenzhou Medical University, Wenzhou, China; ^3^Department of Colorectal and Anal Surgery, The First Affiliated Hospital of Wenzhou Medical University, Wenzhou, China

**Keywords:** double cancer, multiple primary cancers, MPCs, papillary thyroid carcinoma, PTC, lung adenocarcinoma, BRAFV600E, ALK

## Abstract

This report describes a rare case of double primary cancer in a female patient aged 49 years who died 2 years after diagnosis. The patient was diagnosed with BRAFV600E-mutant metastatic papillary thyroid carcinoma (PTC) and *ALK* fusion-positive metastatic lung adenocarcinoma. She presented with multifocal thyroid lesions and underwent radical thyroidectomy and bilateral cervical lymphadenectomy. Thyroid ultrasound revealed the presence of five hypoechoic nodules with irregular margins and microcalcifications; an irregular inhomogeneous hypoechoic level IV cervical lymph node was also found on the right side. Histological analysis confirmed the presence of metastatic PTC, and the tumor tested positive for the BRAFV600E mutation. Ultrasound of the neck, which was performed 4 months postdischarge, revealed enlargement of the left-sided cervical lymph nodes; a biopsy from these nodes confirmed a diagnosis of metastatic PTC. Positron emission tomography-computed tomography scans revealed the presence of multiple pulmonary hypermetabolic foci scattered across bilateral lung fields. Multiple hypermetabolic foci were also observed in the lymph nodes on both sides of the neck, axillae, and mediastinum; in addition, there was evidence of bone destruction with hypermetabolic foci. Supplementary reports from the histological and immunohistochemical analyses of cervical lymph node tissue obtained during primary surgery confirmed the presence of metastatic PTC and poorly differentiated lung adenocarcinoma. In particular, one enlarged cervical lymph node located on the right side of the neck demonstrated tumor components of both PTC and lung adenocarcinoma. Pathological analysis of axillary lymph node puncture biopsy confirmed the presence of metastatic lung adenocarcinoma, and gene analysis revealed the presence of *ALK* fusion. The patient received targeted therapy based on a multidisciplinary discussion. However, she had a poor prognosis and died 2 years after the diagnosis. The initial thyroid ultrasound findings were reviewed retrospectively; the findings suggested that the possibility of double primary cancers should be considered in cases where the enlarged cervical lymph nodes are highly suspicious of PTC and present as inhomogeneous hypoechoic masses with irregular morphology.

## 1 Introduction

Multiple primary cancers (MPCs) refer to two or more primary malignant tumors occurring simultaneously or successively in one or more organs or tissues of the same patient. Warren proposed the following criteria for the diagnosis of MPCs (1): (1) every tumor must be malignant; (2) every tumor has to be confirmed histopathologically; (3) each tumor must occur at different sites and not be continuous with each other; and (4) the possibility of metastatic or recurrent tumors should be eliminated. MPCs are rarely found (2, 3); the five most frequent sites include the breast, liver, head and neck, colorectum, and prostate (2). Unlike the five most frequent sites (2, 3), which account for 3.1% of all MPC diagnoses (2), the thyroid is a rare site for their occurrence.

The increased incidence of MPCs, especially those involving rare thyroid carcinomas, is a major health challenge worldwide. Elucidation of the characteristics of MPCs involving the thyroid may therefore aid in the development of improved management plans (2), improve patient quality of life, and prolong overall survival in these patients. In this report, we describe a rare case of double primary cancers (papillary thyroid carcinoma (PTC) and lung adenocarcinoma) that had a poor prognosis.

## 2 Case description

**Figure 1:**
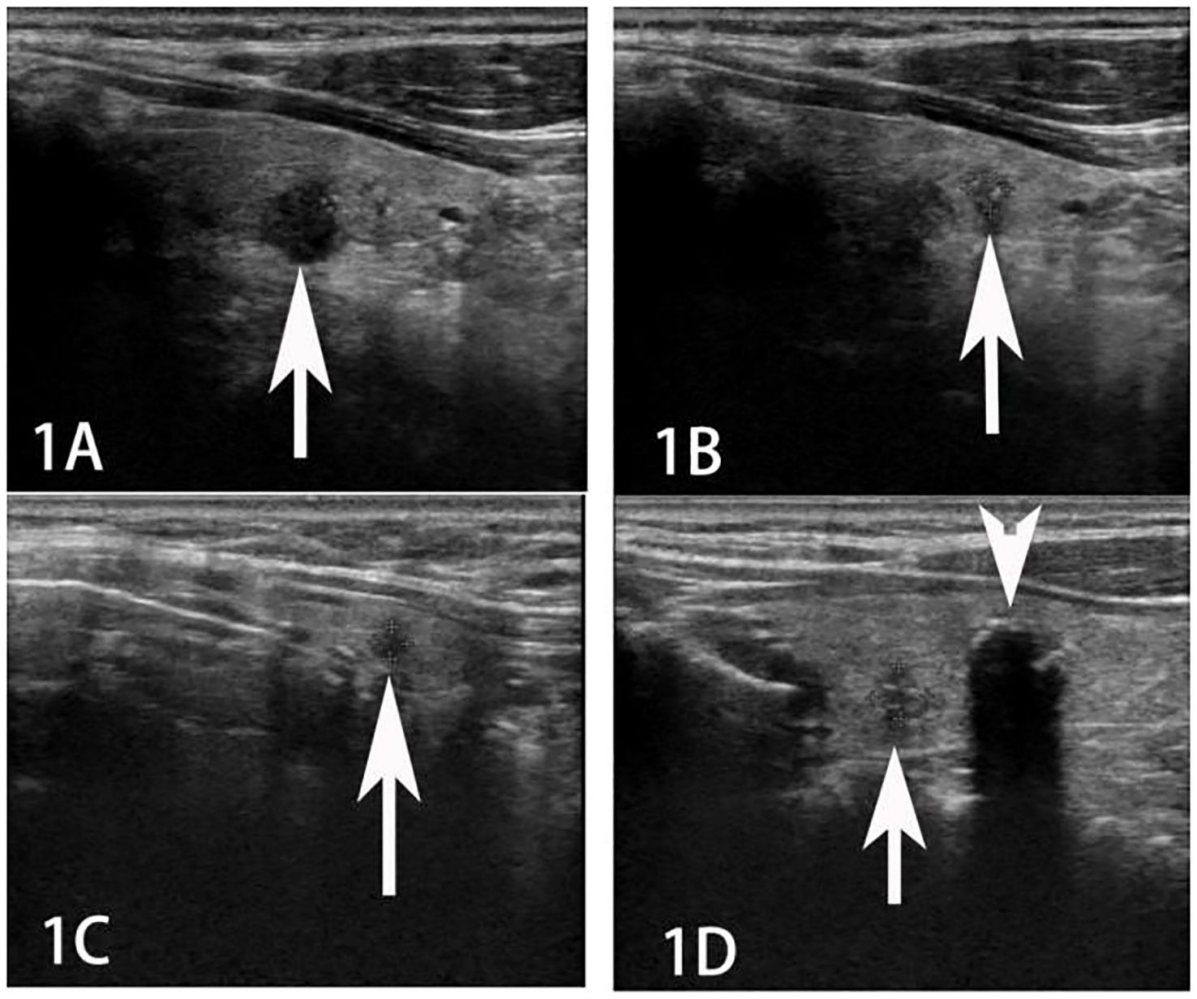
Ultrasound imaging of multiple thyroid cancers (**A, B** tumor in the left lobe; **C** tumor in the isthmus, and **D** tumor in the right lobe; indicated by arrows).

A female patient aged 49 years was admitted for evaluation of thyroid nodules that were detected more than 4 years previously (on 28 August 2018). Thyroid ultrasound (US) revealed the presence of five hypoechoic nodules (two in the inferior pole of the left lobe, two in the middle pole of the right lobe, and one in the isthmus of the thyroid gland) with irregular margins, variable diameters (3–13 mm), and microcalcification (**Figures 1A-D**).

**Figure 2:**
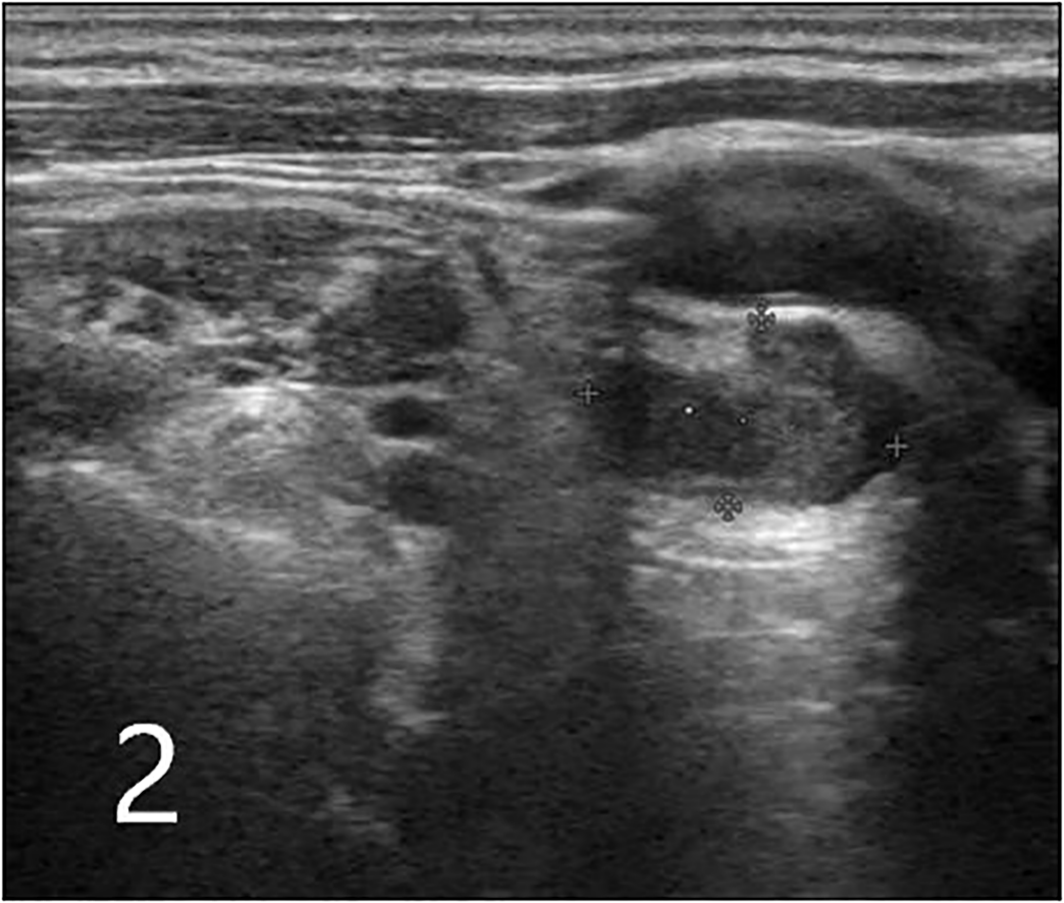
Ultrasound image of an enlarged inhomogeneous hypoechoic cervical lymph node with irregular morphology in level IV on the right side (indicated by arrows; plus sign indicates the measurement of lymph node size, which was 1.5 cm × 0.9 cm).

A highly suspicious irregular inhomogeneous hypoechoic cervical lymph node (size = 1.5 cm × 0.9 cm) was also observed at level IV on the right side (**Figure 2**).

The chest radiograph did not reveal any abnormalities, and the laboratory parameters were within the physiological range. Total thyroidectomy and bilateral cervical lymphadenectomy (levels II, III, IV, V, and VI on the right side and level VI on the left side) were performed. Postoperative histological examination revealed one lesion of PTC and four lesions of papillary thyroid microcarcinoma (PTMC), along with the nodal metastases (stage IVa, T1N1bM0). On gene mutation testing, the PTC tested positive for the BRAFV600E mutation.

**Figure 3:**
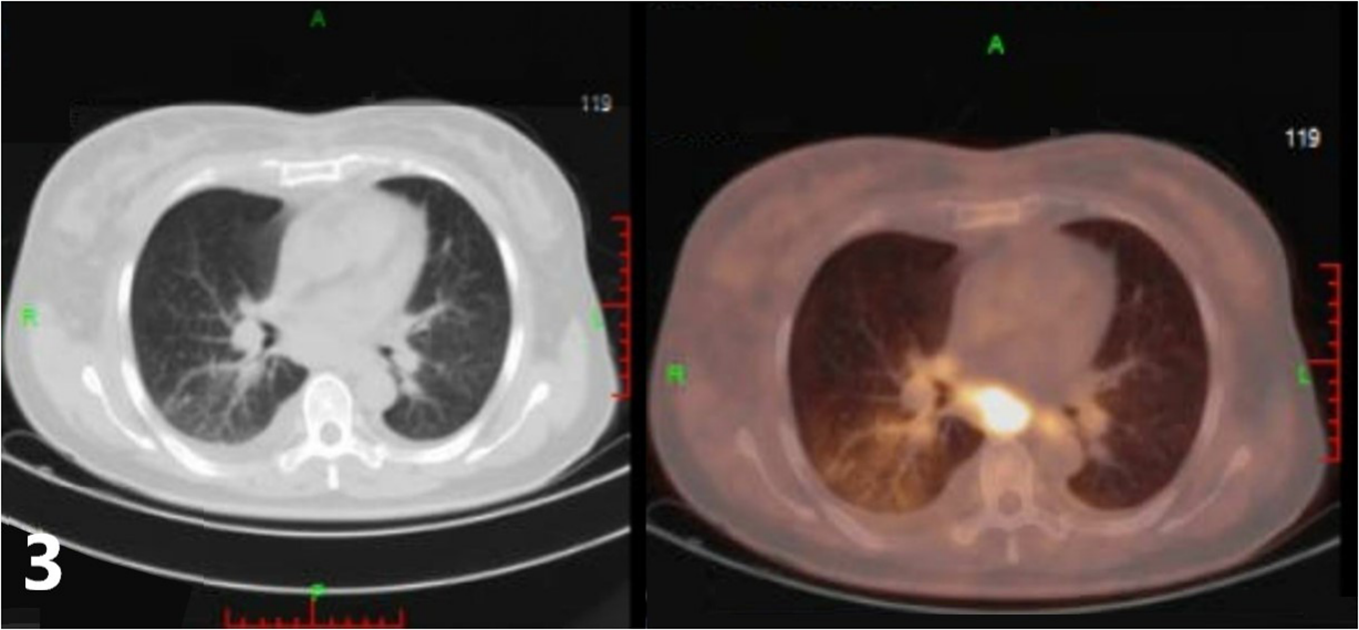
The18-fluorodeoxyglucose positron emission tomography-computed tomography scan (FDG PET/CT) of the lung revealed multiple bilateral pulmonary hypermetabolic foci.

Two weeks after surgery, the patient was hospitalized owing to a neck swelling that was refractory to antibiotic therapy. Neck US revealed bilateral cervical lymphadenopathy that was suspicious of metastatic thyroid cancer. The 18-fluorodeoxyglucose positron emission tomography-computed tomography scan (FDG PET/CT), which was performed for further evaluation, revealed multiple bilateral pulmonary hypermetabolic foci (**Figure 3**, maximum standard uptake value: 4.7) with indistinct borders and multiple bilateral hypermetabolic lymph nodes (maximum size: 24 mm, maximum standard uptake value up to 20) in the neck, bilateral axillae, and mediastinum (stations 2R, 3A, 4R/L, and 5–8); bone destruction with hypermetabolic foci was also observed in the sacrum, right scapula, vertebral arch of L2, and vertebral body of L4. In order to determine whether these lesions represented metastatic thyroid cancer, the sections from the cervical lymph nodes (10 from level VI on the right side, 23 from levels II, III, IV, and V on the right side, and two from level VI on the left side) were evaluated using immunohistochemistry (IHC).

**Figure 4:**
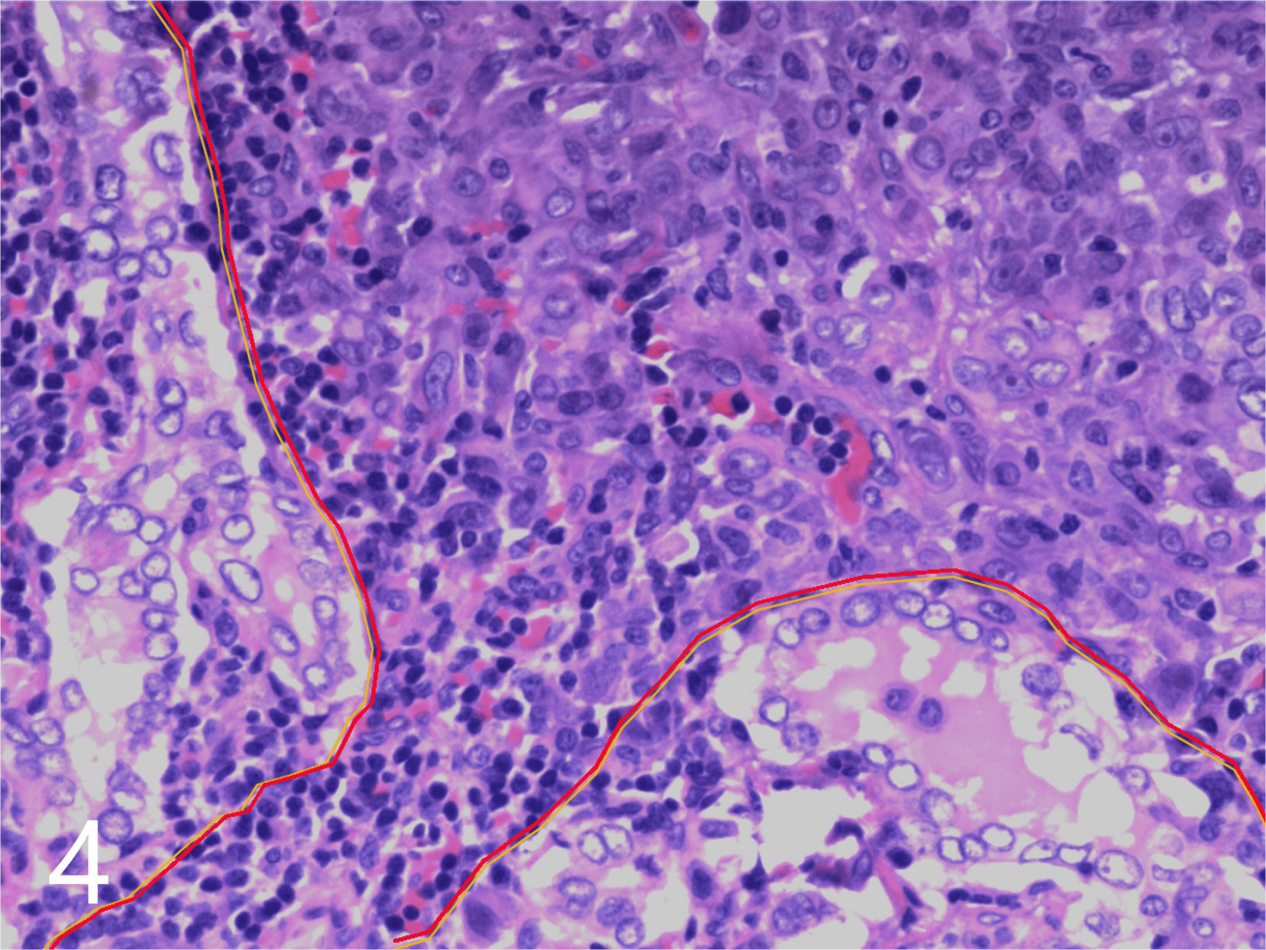
Pathological map of two metastatic tumor components. Thyroid cancer (delineated in yellow on the left and lower side) and lung cancer (delineated in red on the right and upper side) in an enlarged cervical lymph node (hematoxylin and eosin, × 200).

The IHC results from the nodal tissue were as follows: AB (+), CD56 (weakly +), CDX-2 (−), CgA (−), CK (+), CK20 (−), CK5/6 (weakly+), CK7 (+), ER-α (−), GATA3 (−), GCDFP-15 (weakly+), HMB45 (−), Melan-A (−), napsin A (poorly differentiated areas +, glandular duct areas −), P63 (+), PR (−), Sox-10 (−), Syn (−), thyroglobulin (TG) (poorly differentiated areas −, glandular duct areas +), TIF-1 (+), Villin (−), and WT-1 (−). A biopsy of the axillary lymph nodes was considered based on the results. Supplementary reports from the histological and immunohistochemical analyses of cervical lymph nodes (following the first surgery) and the pathological findings from axillary lymph node puncture confirmed the presence of metastatic PTC and lung adenocarcinoma. The IHC findings from the tissue obtained on biopsy of the right axillary lymph nodes were as follows: CK20 (−), CK5/6 (+), CK7 (+), napsin A (+), P63 (+), TIF-1 (+), 34BE12 (+), Ki67 (60%+), TG (−), CDX-2 (−), AB (+), and P40 (weakly +). In particular, components of both malignancies were detected simultaneously in one right-sided cervical lymph node (**Figure 4**).

Gene analysis of axillary lymph node tissue revealed the lesion to be ALK1-positive, EGFR-negative, ROS1-negative, and BRAFV600E-negative.

Following a multidisciplinary discussion, the patient was started on targeted treatment with crizotinib at 5 months postsurgery. Intravenous pamidronate was also administered for the treatment of the bone metastases. Although targeted therapy offered partial tumor response at approximately 6 months, crizotinib resistance developed a year later; she was therefore subsequently treated using alectinib. At 2 years postsurgery, the patient died owing to the development of multiple lesions of primary lung adenocarcinoma. The timetable of the treatment strategy is shown in **Table 1**.

**Table 1:** The timetable of the treatment strategy of the patient.

Date	Chief complaint	Treatment/auxiliary inspection	Diagnosis
30 March 2018	Thyroid nodules detected by US	Total thyroidectomy and bilateral cervical lymphadenectomy/gene testing	Thyroid microcarcinoma with the nodal metastases
21 September 2018	Neck swelling	Antibiotic anti-infection treatment	Exclusion of infection for poor anti-inflammatory effect
22 January–1 February 2019	Neck swelling	Neck US, CT, PET CT, biopsy of the axillary lymph nodes, IHC and gene testing	Double primary malignancies of the lung and thyroid with lymph node metastasis
3 February 2019	Neck swelling	Etotinib (25 mg bid, po)	Double primary cancers
24 March 2019	None	Etotinib (25 mg qd, po), decreased dose by herself	Double primary cancers
24 May 2019	Lumbago	Etotinib (25 mg qod, po), discontinue medication for lumbago	Double primary cancers
30 May 2019	Lumbago	CT, MRI	Double primary cancers with bone metastasis
5 June 2020	Lumbago	Aletinib (4 pieces bid, po) combined with pamidronate (90 mg, iv drip)	Double primary cancers with bone metastasis
29 September 2020	Lumbago	Aletinib (4 pieces bid, po) combined with pemetrexed (80 mg, iv drip)/CT	Double primary cancers with bone metastasis and liver metastasis

## 3 Discussion

Carcinomas of unknown primary origin are defined by the presence of histologically confirmed metastatic carcinomas in the absence of an identifiable primary tumor site, despite extensive multidisciplinary investigations (4). FDG PET/CT scans offer high specificity and sensitivity in the diagnosis of cancer. However, it is difficult to obtain a definite diagnosis of the primary and metastatic tumors in the presence of multiple abnormalities. IHC is a reliable and inexpensive tool for the identification of the site of origin in carcinomas with an unknown primary. In the case described, the diagnosis of PTC had been confirmed; however, biopsies had not been obtained from the multiple bilateral pulmonary lesions. Notably, the presence of multiple abnormalities on the FDG PET/CT scan hinders the identification of the primary site. Although an IHC examination of the cervical lymph nodes was not initially performed in this case, supplemental IHC findings from the cervical and right axillary lymph nodes aided in the determination of the primary site.

In this context, keratins are a family of intermediate filament proteins that are expressed in epithelial cells. Different molecular expression patterns allow for the accurate and elaborate classification of epithelial cells and their neoplasms into different subtypes (5). Among the cytokeratins, CK7 and CK20 have been used most widely to predict the primary site; notably, the CK7+/CK20− expression profile was established in the present case. Complementary organ-specific antibodies, including TTF-1, TG, and napsin A allowed identification of the origin(s) of the two coexisting tumors in the thyroid and lung. TTF-1 and napsin A are used to distinguish primary tumors of the thyroid or lung from those with other tissues of origin. TTF1 is a nuclear transcription factor that promotes embryogenic pulmonary and thyroid differentiation; it is expressed by most, but not all, lung or thyroid neoplasms (6). Although a minority of anaplastic and poorly differentiated thyroid carcinomas may express napsin A, it is also expressed by some lung adenocarcinomas (7).

Primary thyroid carcinomas usually demonstrate TG production; however, primary lung cancers or tumors originating from other sites test negative for this biomarker (8).

In the present case, the tissue specimens from the cervical and axillary lymph nodes were evaluated using IHC staining for CK7, CK20, TTF-1, napsin A, and TG; the specimens from the axillary lymph nodes revealed negative immunoreactivity to CK20 and TG and positive reactivity to CK7, TTF-1, and napsin A. The cervical lymph node specimens included poorly differentiated regions (with positive immunoreactivity to CK7, TTF-1, and napsin A but negative reactivity to CK20 and TG) and glandular regions (with positive immunoreactivity to CK1, TTF-1, and TG but negative reactivity to CK20 and napsin A); one of the cervical lymph nodes obtained from the right side of the neck demonstrated coexistence of both components. These results suggested the presence of double primary carcinomas originating from the thyroid and lung.

Although MPCs are reported to be rare, the morbidity related to these cancers has been gradually increasing. The reported incidence of MPCs varies between 0.73% and 11.7% (9, 10). Depending on the time between diagnosis of the first and second primary tumors, MPCs may be classified as either synchronous (where the second tumor is diagnosed within 6 months of primary cancer) or metachronous (where the second tumor is diagnosed more than 6 months after primary cancer diagnosis) (11). The IHC findings from the cervical and axillary lymph node tissue suggested the presence of synchronous double primary carcinomas originating from the thyroid and lung; the BRAFV600E mutation promoted the development of PTC, while ALK fusion promoted the lung adenocarcinoma. Interestingly, the lymph nodes comprised metastasized cells from both PTC and lung adenocarcinoma. To the best of our knowledge, this is the first study to report the presence of two cancerous components (PTC and lung adenocarcinoma) in a single lymph node; this is an extremely rare finding.

Notably, PTC is the most common malignancy of the thyroid gland; PTMCs are defined as PTCs with diameters of ≤ 10 mm. These tumors (including PTMCs) exhibit an indolent clinical course and are associated with an excellent prognosis. However, a small subset of PTCs may exhibit aggressive phenotypes (including an increased incidence of extrathyroidal extension, lymph node metastases, recurrence, and even death). Unlike other patients with PTC, for whom overtreatment should be avoided, patients with aggressive PTCs should be treated actively. Most current studies focus on the genetic profile (especially the BRAFV600E mutation status) of PTCs to predict tumor behavior; these mutations are prevalent in 40%–70% of PTCs (12) and 57.4% of PTMCs (13). In PTCs (including PTMCs), BRAFV600E mutations are reported to be associated with an increase in aggressive behavior (12).

In this context, thyroid US is the imaging modality of choice for the evaluation of thyroid lesions and the detection of lymph node metastases (12, 14). In the US, PTCs usually manifest as irregularly shaped hypoechoic masses, lesions with microcalcifications, or masses with an aspect ratio of > 1 (15). In cases with cervical lymph node metastases, multiple lesions are more commonly found than single lesions. In the present case, the lesions exhibited more than one of the malignant features mentioned above. Cervical metastases from PTC usually manifest as round, cystic, or microcalcified cervical nodules in the US (16, 17). One cervical lymph node in the present case appeared as an irregular inhomogeneous hypoechoic mass on initial US examination. These features are in contrast to those of the classical presentation of PTC-related metastases.

*ALK*, which encodes a receptor-type tyrosine kinase, is considered to be the driver gene for tumorigenesis. *ALK* fusion occurs in cases of breakage and fusion with other genes, with the fusion between *EML4* and *ALK* being the most important. The *EML4-ALK* fusion gene, which is found in 3%–5% of non-small cell lung cancer cases (18) and occasionally in PTC (19), encodes the EML4-ALK fusion protein that can directly form an ALK dimer. The dimer subsequently activates the ALK and downstream signaling pathways, including the RAS/ERK/STAT3/mTOR or BRAF pathways, and contributes to carcinogenesis in non-small cell lung cancer (20, 21). The carcinogenic fusion of EML4-ALK mostly occurs in female patients with non-small cell lung cancer who have no prior history of smoking (22). In the present case, the patient was a woman and had no history of smoking; she was asymptomatic and was diagnosed and treated after the detection of thyroid nodules during physical examination. Investigations revealed the presence of cervical and axillary lymphadenopathy and bone destruction. Various targeted therapeutic drugs have been developed in recent years for treating cases with EML4-ALK fusions. Crizotinib, which was the first targeted therapy drug to be used, is more effective than traditional standard chemotherapy (23, 24). It offers an objective response rate of 53% with a mean progression-free survival of 8.5 months; however, resistance to crizotinib usually develops within 1 year of treatment. Second-generation EML4-ALK-targeted drugs (such as ceritinib, brigatinib, and alectinib), the third-generation targeted drug lorlatinib, and the fourth-generation targeted drug repotrectinib (TPX-0005) have been developed to overcome crizotinib resistance (20). Drug resistance was also observed in the study case, necessitating an alteration of the therapeutic regimen. The patient died 20 months after initiation of targeted drug therapy; the poor prognosis in this case was consistent with findings from previous studies (2, 3, 25), which reported poor outcomes in young female patients with MPCs.

A review of the initial cervical US images revealed only one unique finding: an enlarged cervical lymph node, which appeared as an irregular hypoechoic nodule. This differed from the usual features (round shape, microcalcification, or cystic change) of PTC-related metastases. In this context, it is essential to suspect the presence of metastases from other malignant tumors in cases of PTC where the US reveals enlarged inhomogeneous hypoechoic and irregular cervical lymph nodes; alternatively, this finding may indicate the coexistence of metastases from PTC and malignant tumors of different origins (suggesting the occurrence of double primary cancers). Postoperative pathological evaluation should preferably be further supported by IHC and gene testing to confirm the origin of the metastasis. This may enable early accurate diagnosis and comprehensive treatment.

## 4 Conclusions

Although biopsies could not be obtained from the lung lesions (in view of the retrospective evaluation of the present case), IHC examination of the cervical and right axillary lymph nodes identified the primary sites to be the thyroid and lung. Despite these limitations, this report describes a rare case with double primary cancers (BRAFV600E-mutant metastatic PTC and ALK fusion-positive metastatic lung adenocarcinoma) that had a poor prognosis.

To the best of our knowledge, this is the second report on the diagnosis of concomitant pulmonary and thyroid primary adenocarcinomas using FDG PET/CT and IHC. The cervical lymph node metastases comprised both PTC and lung adenocarcinoma components. In particular, the tumor components of PTC and lung adenocarcinoma were detected in one cervical lymph node from level VI; this is the first report to describe these findings. The mentioned cervical lymph node presented as an irregular inhomogeneous hypoechoic mass during the initial thyroid US examination. These features differed from the typical features of metastases from PTCs. Radiologists and clinicians therefore need to be aware of these US features in enlarged cervical lymph nodes, as they may indicate the presence of metastases from double primary cancers. Additional histological examination, immunostaining, and gene testing may be needed to establish an accurate diagnosis and facilitate treatment earlier in the course of the disease.

## Data availability statement

The original contributions presented in the study are included in the article/supplementary material. Further inquiries can be directed to the corresponding author.

## Ethics statement

The studies involving humans were approved by The Ethics Committee in Clinical Research of the First Affiliated Hospital of Wenzhou Medical University. The studies were conducted in accordance with the local legislation and institutional requirements. Written informed consent for participation in this study was provided by the participants’ legal guardians/next of kin. Written informed consent was obtained from the individual(s) for the publication of any potentially identifiable images or data included in this article.

## Author contributions

Data collection and analysis: PL and Y-FP. Writing—original draft and literature research: S-PC and XJ. Writing—review and editing: XJ. All authors contributed to the article and approved the submitted version.
